# An Anti-Human ICAM-1 Antibody Inhibits Rhinovirus-Induced Exacerbations of Lung Inflammation

**DOI:** 10.1371/journal.ppat.1003520

**Published:** 2013-08-01

**Authors:** Stephanie Traub, Alexandra Nikonova, Alan Carruthers, Rebecca Dunmore, Katherine A. Vousden, Leila Gogsadze, Weidong Hao, Qing Zhu, Katie Bernard, Jie Zhu, Michael Dymond, Gary R. McLean, Ross P. Walton, Nicholas Glanville, Alison Humbles, Musa Khaitov, Ted Wells, Roland Kolbeck, Andrew J. Leishman, Matthew A. Sleeman, Nathan W. Bartlett, Sebastian L. Johnston

**Affiliations:** 1 National Heart and Lung Institute, MRC and Asthma UK Centre in Allergic Mechanisms of Asthma, Centre for Respiratory Infection, Imperial College London, London, United Kingdom; 2 MedImmune Ltd, Cambridge, United Kingdom; 3 MedImmune LLC, Gaithersburg, Maryland, United States of America; 4 AstraZeneca, Charnwood, United Kingdom; 5 NRC Institute of Immunology FMBA, Moscow, Russia; Johns Hopkins University - Bloomberg School of Public Health, United States of America

## Abstract

Human rhinoviruses (HRV) cause the majority of common colds and acute exacerbations of asthma and chronic obstructive pulmonary disease (COPD). Effective therapies are urgently needed, but no licensed treatments or vaccines currently exist. Of the 100 identified serotypes, ∼90% bind domain 1 of human intercellular adhesion molecule-1 (ICAM-1) as their cellular receptor, making this an attractive target for development of therapies; however, ICAM-1 domain 1 is also required for host defence and regulation of cell trafficking, principally via its major ligand LFA-1. Using a mouse anti-human ICAM-1 antibody (14C11) that specifically binds domain 1 of human ICAM-1, we show that 14C11 administered topically or systemically prevented entry of two major groups of rhinoviruses, HRV16 and HRV14, and reduced cellular inflammation, pro-inflammatory cytokine induction and virus load *in vivo*. 14C11 also reduced cellular inflammation and Th2 cytokine/chemokine production in a model of major group HRV-induced asthma exacerbation. Interestingly, 14C11 did not prevent cell adhesion via human ICAM-1/LFA-1 interactions *in vitro,* suggesting the epitope targeted by 14C11 was specific for viral entry. Thus a human ICAM-1 domain-1-specific antibody can prevent major group HRV entry and induction of airway inflammation *in vivo*.

## Author Summary

Viruses exploit receptors on the host cell to cause infection. Therapies aimed at blocking virus-receptor interactions have the potential to prevent viral disease. Cellular receptors are also important for normal host cell function. Therefore, new therapies targeting these receptors to block viral infection may also inadvertently alter the physiology of the host cell. Viral pathogens, such as the cold virus (rhinovirus), are believed to be the major cause of asthma attacks and exacerbations in chronic obstructive pulmonary disease (COPD). In this study, we show that it is possible to identify novel therapeutic antibodies that block infection with rhinovirus without impairing the receptors' main function of cell adhesion. We then use animal models that show that an antibody can inhibit virus-induced lung inflammation and disease. Moreover, we show that this antibody can also inhibit a virally induced asthma exacerbation. This work is relevant in that it shows that antibodies can be tailored to distinct regions of viral receptors to block infection without inhibiting the receptors' normal cellular function. This is important for the development of new treatments that will prevent diseases caused by infection with rhinovirus, such as exacerbations of asthma and COPD.

## Introduction

Human rhinoviruses (HRVs) infect the upper respiratory tracts of healthy subjects and cause around three quarters of common colds [1], [2]. Recently, it has become clear that HRVs are also major causes of severe, life-threatening illnesses in susceptible populations: 50–85% of acute asthma exacerbations are now known to be caused by HRV infections across all age groups [3], [4], [5], [6]. Further, wheezing illnesses in infancy caused by HRVs are strongly associated with a very high risk of later development of childhood asthma [7], [8], [9]. HRV infections are also associated with the majority of acute exacerbations of chronic obstructive pulmonary disease (COPD) [10], [11] and cystic fibrosis [12] and cause life-threatening illnesses in susceptible populations such as infants [13], the elderly [14] and immuno-compromised individuals [15]. Against this background, there is an urgent need to develop effective medications to combat HRV induced illnesses. To date there are no licensed treatments or vaccinations.

Rhinoviruses are positive-stranded RNA viruses of the Picornaviridae family and more than 100 antigenically distinct serotypes have been identified [1], [16], [17]. Serotyped strains of HRVs are classified by use of their entry receptor into minor group viruses, which comprise ∼10% of identified serotypes and use low-density-lipoprotein receptor and related molecules [17] and major group viruses, comprising ∼90% of identified serotypes, which use intercellular adhesion molecule 1 (ICAM-1, CD54) as their receptor [2], [16]. Thus ICAM-1 is an attractive target for development of new therapies to combat major group HRV infections.

However, apart from its role as HRV receptor, ICAM-1 also has important host functions and in particular it is critical in host defence against numerous pathogens via its role in leukocyte recruitment and activation [18], [19], [20]. Thus inhibiting the function of ICAM-1 has potential to interfere not only with HRV cell binding, but also to impair host defence by inhibiting its interaction with its natural host ligands. ICAM-1 is a member of the immunoglobulin (Ig) superfamily and the extracellular portion of the molecule has five Ig-like domains termed D1 as the most distal, to D5 as the most proximal to the cell membrane. HRV serotypes binding ICAM-1 have been reported to bind only to D1, while LFA-1 and Mac-1 bind to domains D1 and D3, respectively [21], [22], [23]. Thus antibodies specific to D1 of ICAM-1 would be needed to interfere with HRV infection; however they could potentially interfere with ICAM-1/LFA-1 interactions.

In the present report we characterise an anti-human ICAM-1-specific antibody (14C11) and show its binding is specific to human ICAM-1 D1, that it is inhibitory against multiple major group HRV serotypes *in vitro*, and prevents major group HRV infection and HRV induced exacerbation of allergic airway inflammation *in vivo*. We also show that this antibody does not inhibit human ICAM-1/LFA-1 interactions and therefore should not interfere with cellular trafficking.

## Results

### 14C11 binds in domain 1 of human ICAM-1, does not bind to mouse ICAM-1 and does not inhibit human ICAM-1/LFA-1 interaction

**Figure 1:**
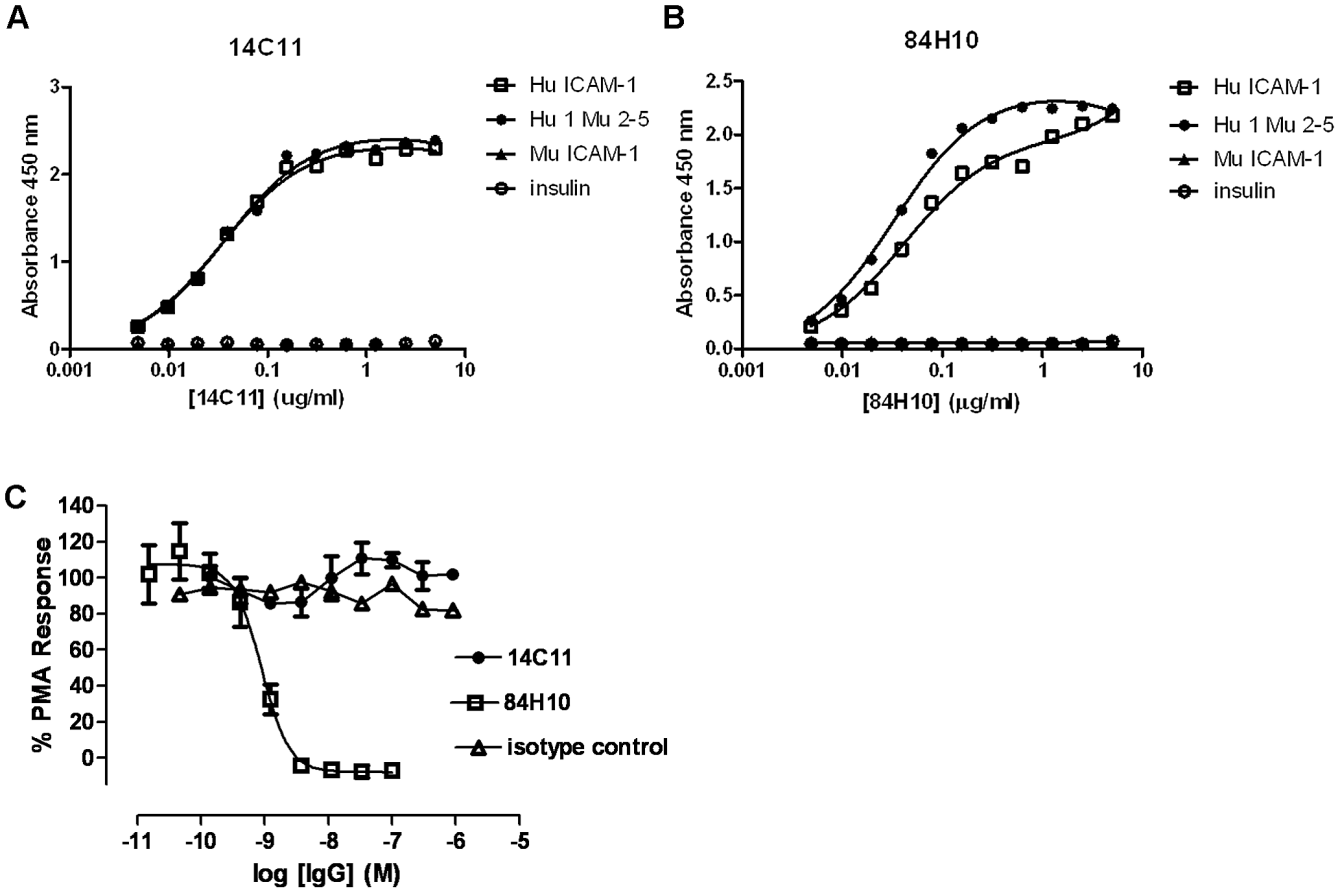
14C11 binds in domain 1 of human ICAM-1, does not bind to mouse ICAM-1 and does not inhibit human ICAM-1/LFA-1 interaction. (A)14C11 and (B) 84H10 antibody were tested for binding of antigens (human ICAM-1 (Hu ICAM-1), mouse ICAM-1 (Mu ICAM-1) and a recombinant protein consisting of human ICAM-1 D1 and mouse ICAM-1 D2-5 (Hu1 Mu2-5)). Jurkat E6.1 cells were labelled with Calcein-AM dye. Serial dilutions of the antibodies 14C11, 84H10 and isotype control were added in an ICAM-1-Fc coated plate. (C) PMA and labelled Jurkat E6.1 cells were added into the plate. Binding to LFA-1 was determined by fluorescein detection. Data were analysed by normalising values to no PMA (0% signal) and PMA no antibody (100% signal) controls. Data are a representative of three experiments for (A) and (B) and four to seven experiments for (C). Data are expressed as mean (± SEM).

ICAM-1 plays an important role in trans-endothelial migration of leukocytes and activation of T cells via LFA-1. In addition it is also the entry receptor for major group HRVs. We were interested in identifying antibodies suitable to inhibit HRV-ICAM-1 interactions for the potential treatment of HRV-induced exacerbations in airway disease without compromising the host leukocyte trafficking by interference of ICAM-1 interactions with the integrin LFA-1. We thus initially investigated the binding properties of the commercially available antibodies to human ICAM-1 - 14C11 and 84H10 - to determine whether they bind to domain 1 of human ICAM-1 (Hu ICAM-1) the putative binding site for viral entry [2], [16]. Biotinylated Hu ICAM-1, a chimeric protein consisting of domain 1 of human ICAM-1 and domains 2–5 of mouse ICAM-1 (Hu1 Mu2-5), murine ICAM-1 (Mu ICAM-1) and insulin as a control protein were incubated with 14C11 or 84H10 to test their binding specificity. We found binding of 14C11 and 84H10 to both Hu ICAM-1 and Hu1 Mu2-5 confirming that 14C11 binds specifically to domain 1 of human ICAM-1, but not to murine ICAM-1 (Figure 1A and 1B). Additionally, cells were stimulated with PMA in a human T cell (Jurkat cell) adhesion assay, to determine whether 14C11 or 84H10 could inhibit human ICAM-1/LFA-1 interactions. Neither 14C11 nor the isotype control was able to prevent cell adhesion via LFA-1 whereas the anti-ICAM-1 antibody 84H10 blocked the ICAM-1/LFA-1 interaction and failed to retain the labelled Jurkat cells (Figure 1C). These results emphasise that the domain specificity of 14C11 suggests it will not block human ICAM-1/LFA-1 interaction. Regarding binding to other members of the immumoglobulin (Ig) superfamily structurally related to huICAM-1 the specification provided by the manufacturer stated that 14C11 showed no cross-reactivity with rabbit ICAM-1, human ICAM-2, human ICAM-3, human VCAM-1, and ‘mouse DCC’. We also demonstrated the binding specificity of 14C11 observing binding to human ICAM-1 and a chimeric mouse ICAM-1 with domain 1 substituted for human ICAM-1 Ig domain 1. In the same assay no binding to mouse ICAM-1, human ICAM-2, human ICAM-3, human ICAM-5 or human VCAM-1 was observed. (Fig. S1).

### The anti-ICAM-1 antibody inhibits major group HRV replication *in vitro*

**Figure 2:**
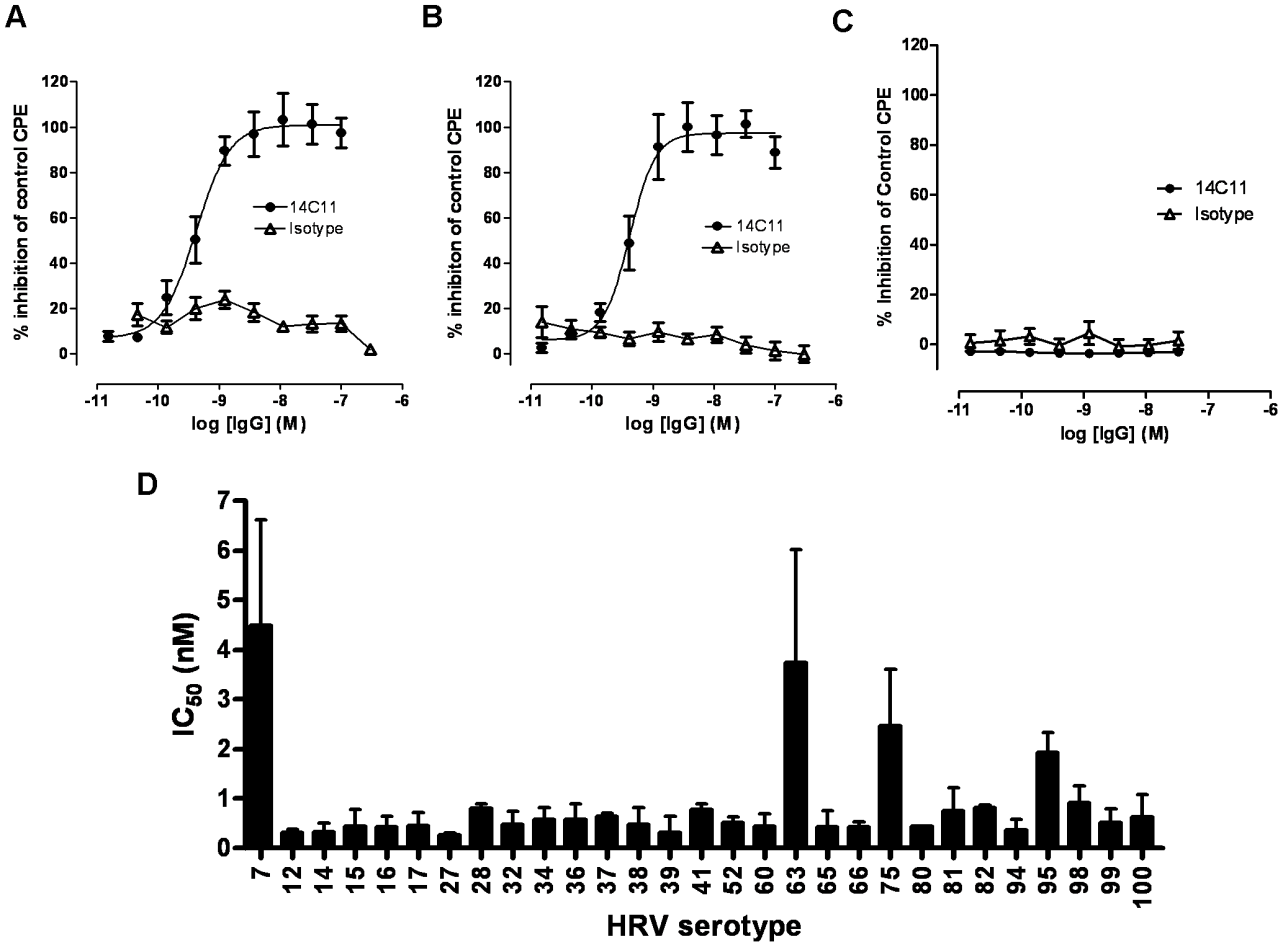
14C11 inhibits major group HRV replication *in vitro*. Ohio HeLa cells were pre-incubated with serial dilutions of 14C11 or isotype control and infected with (A) HRV16, (B) HRV14 and (C) minor group HRV25. The antiviral effect of 14C11 was determined by CPE reduction assay and expressed as % of control. (D) IC_50_s for 14C11 were determined for major HRVs by CPE assay as indicated in. Experiment (A), (B) and (C) were performed in sextuplicates and (D) is a pool of two independent experiments. Data are expressed as mean (± SEM).

Next we tested the effect of 14C11 in an *in vitro* HRV infection assay. Using a cytopathic effect assay (CPE) we showed the antiviral effect of 14C11 in inhibiting the replication of HRV16 (Figure 2A) and HRV14 (Figure 2B) with increasing concentration of antibody. 14C11 did not inhibit CPE caused by infection with minor group serotype HRV25 (Figure 2C) consistent with ICAM-1 blockade-specific mechanism of action. Isotype control antibody did not prevent replication of HRV16 or HRV14 in HeLa Ohio cells. Additionally, the median tissue culture inhibitory concentration (IC_50_) was assessed for a wide range of major group HRVs as indicated in Figure 2D, suggesting a consistent prevention of replication by 14C11 for many major type HRVs, the great majority with sub-nanoMolar IC_50_s. These results emphasise that the domain specificity of 14C11 indicates it inhibits human ICAM-1-HRV binding but does not block human ICAM-1/LFA-1 interaction.

### The anti-ICAM-1 antibody 14C11 inhibits major group HRV induced inflammation and lung virus RNA levels *in vivo*

**Figure 3:**
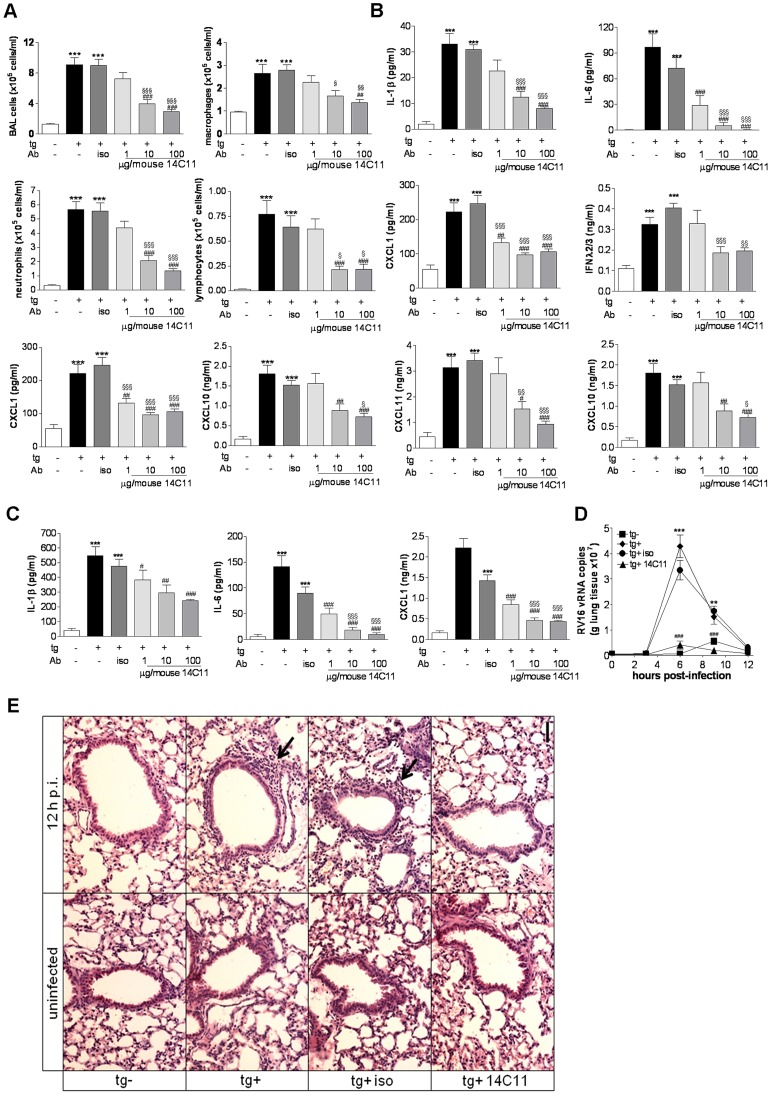
14C11 antibody inhibits major group HRV16 infection *in vivo*. Groups of 7 mice were dosed intranasally with 14C11 or isotype control 2 hours prior to intranasal infection with HRV16. (A) Total BAL cells, macrophages, lymphocytes and neutrophils were assessed with differentially cytospin counts day 2 after infection. (B) Levels of proinflammatory cytokines and chemokines IL-1β, IL-6, CXCL1, IFNλ2/3, CXCL11 and CXCL10 in cell-free BAL were determined by MSD or quantitative ELISA 2 days after infection. (C) Proinflammatory cytokines and chemokines IL-1β, IL-6 and CXCL1 in lung homogenate were assessed with MSD 2 days after infection. (D) Groups of 3–5 mice were dosed intranasally with 14C11 or isotype control 2 hours prior to intranasal infection with HRV16 and vRNA in lung tissue was assessed by qPCR at the timepoints indicated. (E) Inflammatory cells in the lungs prior to infection (uninfected) and at 12 hours post-infection (12 p.i.) of huICAM negative (tg−) and huICAM-expressing (tg+) mice without antibody treatment, or for tg+ mice following isotype control antibody (tg+ iso) or 14C11 antibody treatment (tg+ 14C11) assessed by H&E staining Arrows indicate peribronchial cellular inflammation. Data are expressed as mean (± SEM). Significance was assessed by One-way ANOVA test with Bonferroni's Multiple Comparison test as post-test. ***p<0.001 and **p<0.01 vs HRV16 infected transgenic negative mice; ^#^p<0.05, ^##^p<0.01 and ^###^p<0.001 vs HRV16 infected transgenic positive mice. Data are representative of 3 independent experiments each.

To test the effect of the anti-ICAM-1 antibody 14C11 in an *in vivo* major group HRV infection model, we challenged transgenic Balb/c mice over-expressing extracellular domain 1 and 2 of human ICAM-1 (tg+) and non-transgenic littermates (tg−) with major group HRV16 as described previously [24]. Transgenic positive (tg+) mice were dosed 2 hours prior to viral challenge with 3 doses of 14C11 intranasally; 1 µg, 10 µg and 100 µg/mouse 14C11 or 100 µg/mouse isotype control and were subsequently infected intranasally with major group HRV16. Transgenic negative (tg−) mice were challenged with the same dose of HRV16 as the tg+ littermates. BAL was performed 2 days after infection. Tg+ mice showed a robust cell infiltration and cytokine and chemokine production in the lung compared to tg− mice for all parameters measured. Isotype control treatment did not alter HRV induced airway inflammation for any endpoint (Figure 3). Antibody treatment in the absence of infection did not cause any measurable immune response in the lung (data not shown). 14C11 significantly reduced total BAL cell, macrophage, neutrophil and lymphocyte numbers in a dose dependent manner (Figure 3A). HRV induction of the pro-inflammatory cytokines IL-1β and IL-6, the chemokines CXCL1, CXCL11 and CXCL10, as well as type III interferon IFNλ2/3 were all significantly reduced in BAL by 14C11 treatment (Figure 3B). Induction of IL-1β, IL-6 and CXCL1 were also significantly reduced in lung homogenate (Figure 3C). The kinetics of HRV16 viral load was assessed by real-time PCR. Tg+ mice and tg+ mice pre-treated with isotype control showed increased HRV16 vRNA levels at 6 to 9 hours after challenge compared to tg− mice, while the HRV16 vRNA levels were completely abolished by 14C11 treatment (Figure 3D). H&E staining of lung sections from tg+ mice infected with HRV or isotype pre-treated HRV infected mice showed increased inflammatory cells in the lung. However, in 14C11 treated animals as well as control tg− animals challenged with HRV only marginal inflammation was observed (Figure 3E). The human ICAM-1 specific antibody 14C11 could therefore prevent HRV16 entry and replication as well as induction of airway inflammation *in vivo*.

To investigate further how 14C11 influences airway inflammation induced by another major group virus, we analysed BAL from HRV14 infected mice. We chose this serotype as HRV14 is in the HRV-B type group and is genotypically distinct from HRV16 (which belongs to the HRV genotype group A). BAL cell analysis revealed increases in total BAL cell, neutrophil and lymphocyte numbers in HRV14 infected tg+ mice and HRV14 infected tg+ mice pre-treated with isotype control compared to tg− controls. Virus induction of total BAL cell, neutrophil and lymphocyte numbers were significantly reduced in tg+ mice pre-treated with 14C11 (Figure S1A). The induction of proinflammatory cytokine IL-6, the chemokines CXCL1, CXCL11 and CXCL10 as well as type III interferon IFNλ2/3 were not different in tg+ mice infected with HRV14 and isotype pre-treated tg+ infected mice. However, induction of cytokines, chemokines and type III interferon IFNλ2/3 in both BAL (Figure S1B) and lung homogenate (Figure S1C) were reduced by 14C11 treatment in HRV infected tg+ mice, to levels similar to those in HRV14 challenged tg− control mice. Thus 14C11 is able to inhibit group B HRV-as well as group A HRV-induced inflammation.

### Systemic administration of the anti-ICAM-1 antibody 14C11 is protective against HRV16-induced airway inflammation *in vivo*

**Figure 4:**
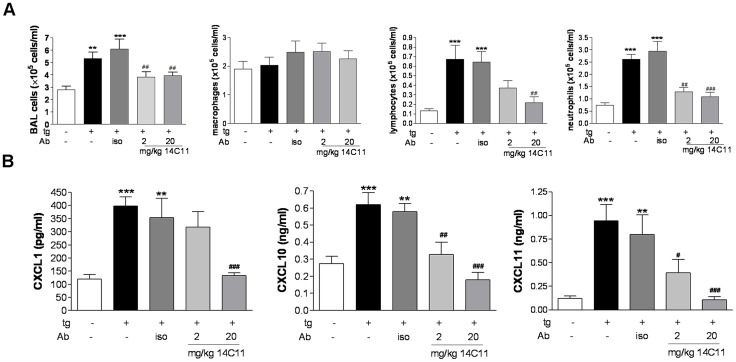
Effect of systemically dosed 14C11 antibody on HRV16 infection in vivo. Mice were dosed intravenously with 14C11 24 hours prior to intranasal infection with HRV16 (n = 9 for tg− group; n = 6 for tg+ groups). (A) Total BAL cells, amacrophageslymphocytes and neutrophils were assessed by cytospin 2 days after infection. (B) The chemokines CXCL1, CXCL11 and CXCL10 in BAL were determined by MSD or quantitative ELISA 2 days after infection. Data are expressed as mean (± SEM). Significance was assessed by One-way ANOVA test with Bonferroni's Multiple Comparison test as post-test. **p<0.01 and ***p<0.001 vs HRV16 infected transgenic negative mice; ^#^p<0.05, ^##^p<0.01 and ^###^p<0.001 vs HRV16 infected transgenic positive mice; Data are representative of 3 independent experiments.

As therapeutic antibodies for the treatment of respiratory diseases are typically dosed parenterally [25] we next evaluated the pharmacological activity of 14C11 via the intravenous route of administration. Mice were pre-treated 24 hours prior to viral challenge and then cell influx and pro-inflammatory cytokine release were assessed. Control groups of HRV16 infected tg+ and isotype treated control mice showed elevated levels of total BAL cells, lymphocytes and neutrophils on day 2 after infection compared to HRV16 challenged tg− mice (Figure 4A).). Administration of intravenous 14C11 24 h prior to HRV infection dose dependently reduced airways inflammation in terms of total numbers of BAL cells, lymphocytes and neutrophils. (Figure 4A). 14C11 significantly reduced in a dose-dependent manner the induction of CXCL1, CXCL11 and CXCL10 in the BAL of tg+ HRV16 infected mice compared to untreated or isotype pre-treated HRV16 infected tg+ mice (Figure 4B). Induction of IL-1β and IL-6 as well as IFNλ2/3 was also reduced by 14C11 treatment in tg+ HRV16 infected mice (Figure S2A). Similar results were found in lung homogenate for protein levels of IL-1β, IL-6 and CXCL1 (Figure S2B). Systemic administration of 14C11 is therefore protective against HRV16 infection in terms of induction of airway inflammation *in vivo*.

### 14C11 does not influence inflammation induced via mechanisms independent of human ICAM-1 *in vivo*

**Figure 5:**
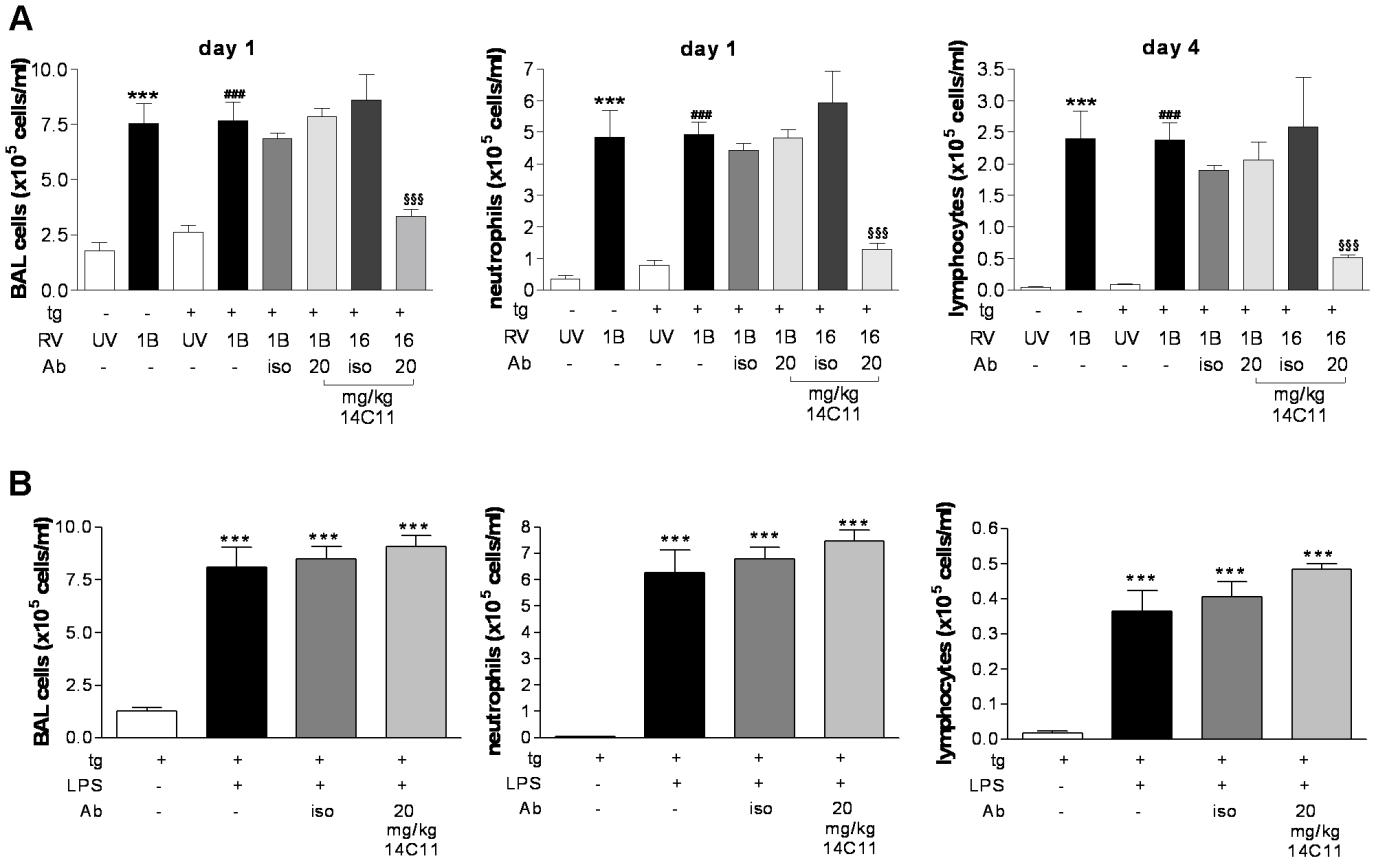
14C11 does not inhibit inflammation induced via mechanisms independent of human ICAM-1. Mice were dosed intravenously with 14C11 24 hours prior to intranasal dosing with HRV1B, UV-inactivated HRV1B (UV) or HRV16 (n = 4 for tg− UV, tg− 1B, tg+ 16 iso and tg+ 16 14C11 groups; n = 6 for tg+ UV, tg+ 1B, tg+ 1B iso and tg+ 1B 14C11 groups). (A)Total BAL cells, neutrophils (on day 1) and lymphocytes (on day 4) were assessed with differentially stained cytospins. Data expressed as mean (± SEM). Significance was assessed by One-way ANOVA test with Bonferroni's Multiple Comparison test as post-test. ***p<0.001 vs UV-RV1B in transgenic negative mice; ^###^p<0.001 vs UV-RV1B in transgenic positive mice; ^§^p<0.05 and ^§§§^p<0.001 vs isotype treated HRV16 infected transgenic positive mice. Data are representative of 2 independent experiments. (B) Groups of 7 mice were dosed intravenously with 14C11 24 hours prior to intranasal dosing with 1 µg LPS/mouse. Total BAL cells, lymphocytes and neutrophils were assessed with differentially stained cytospins day 1 after infection. Data are expressed as mean (± SEM). Significance was assessed by One-way ANOVA test with Bonferroni's Multiple Comparison test as post-test. *p<0.05, **p<0.01 and ***p<0.001 vs transgenic positive mice without treatment. Data are representative of 2 independent experiments.

Antibodies are large complex proteins that not only bind antigen via the variable domain but also bind Fc receptors on antigen presenting cells by binding via the constant domain. Moreover 14C11 is a mouse anti-human IgG1 type antibody and could potentially bind to mouse Fcγ receptors on innate immune cells, such as FcγRIIB, providing a potential inhibitory signal. To investigate whether the 14C11 antibody non-specifically inhibited inflammation by cross-linking human ICAM-1 and binding Fcγ receptor cells we therefore investigated whether 14C11 could inhibit cellular inflammation evoked in tg+ mice, but by a non human ICAM-1 mediated infection using minor group HRV1B infection. Transgenic negative and transgenic positive littermates were dosed intravenously 24 hours prior to viral infection with or without 20 mg/kg isotype control or 14C11 antibody and consequently infected with UV-inactivated HRV1B or with HRV1B. Significant induction of total BAL cells and neutrophils at day 1 post infection and lymphocyte numbers at day 4 could be observed compared to mice infected with UV-inactivated HRV1B in both types of mice (Figure 5A). Induction by HRV1B of each cell type was virtually identical in tg+ and tg− mice, suggesting that the transgenic over-expression of the chimeric human/mouse ICAM-1 molecule did not influence cell trafficking via potential interactions with mouse LFA-1.

Pre-treatment with isotype control or 14C11 antibody in transgene positive mice infected with HRV1B did not alter the induction of cellular inflammation, while infiltration of inflammatory cells in the lung of HRV16 infected transgene positive mice was significantly reduced by 14C11, showing that 14C11 was able to block HRV entry and replication in the same experiment (Figure 5A and S3A). Similarly, 14C11 administration did not reduce HRV1B-induction of CXCL1, CXCL10 or CXCL11, nor the cytokines IL-1β, IL-6 or IFNλ2/3 in BAL or lung homogenate (Figure S3B and C).

We also explored whether 14C11 would non-specifically inhibit inflammatory responses using an LPS challenge model in the presence or absence of 14C11 antibody. Although we would not expect 14C11 to interfere with mouse LFA-1 - mouse ICAM-1 interaction it was still necessary to demonstrate the specificity of 14C11 by performing studies in a model of airway inflammation that did not require human ICAM-1. Mice challenged with LPS, LPS and systemic administered isotype control or 14C11 showed significant increases in total BAL cells on day 1 and neutrophil and lymphocyte numbers on day 4 after infection (Figure 5B), which were not altered by pre-treatment with 14C11. In addition, pro-inflammatory cytokines were also unaffected by 14C11 treatment (Figure S3D and E).

Taken together, this indicates that the human ICAM-1 specific antibody 14C11 could prevent airway inflammation *in vivo* for two major group HRVs, HRV16 and HRV14, but had no non-specific effects on inflammation induced by a stimuli involving human ICAM-1 independent mechanisms.

### Sustained effect of a single dose of the anti-ICAM-1 antibody 14C11 on inhibition of HRV16 induced airway inflammation *in vivo*

**Figure 6:**
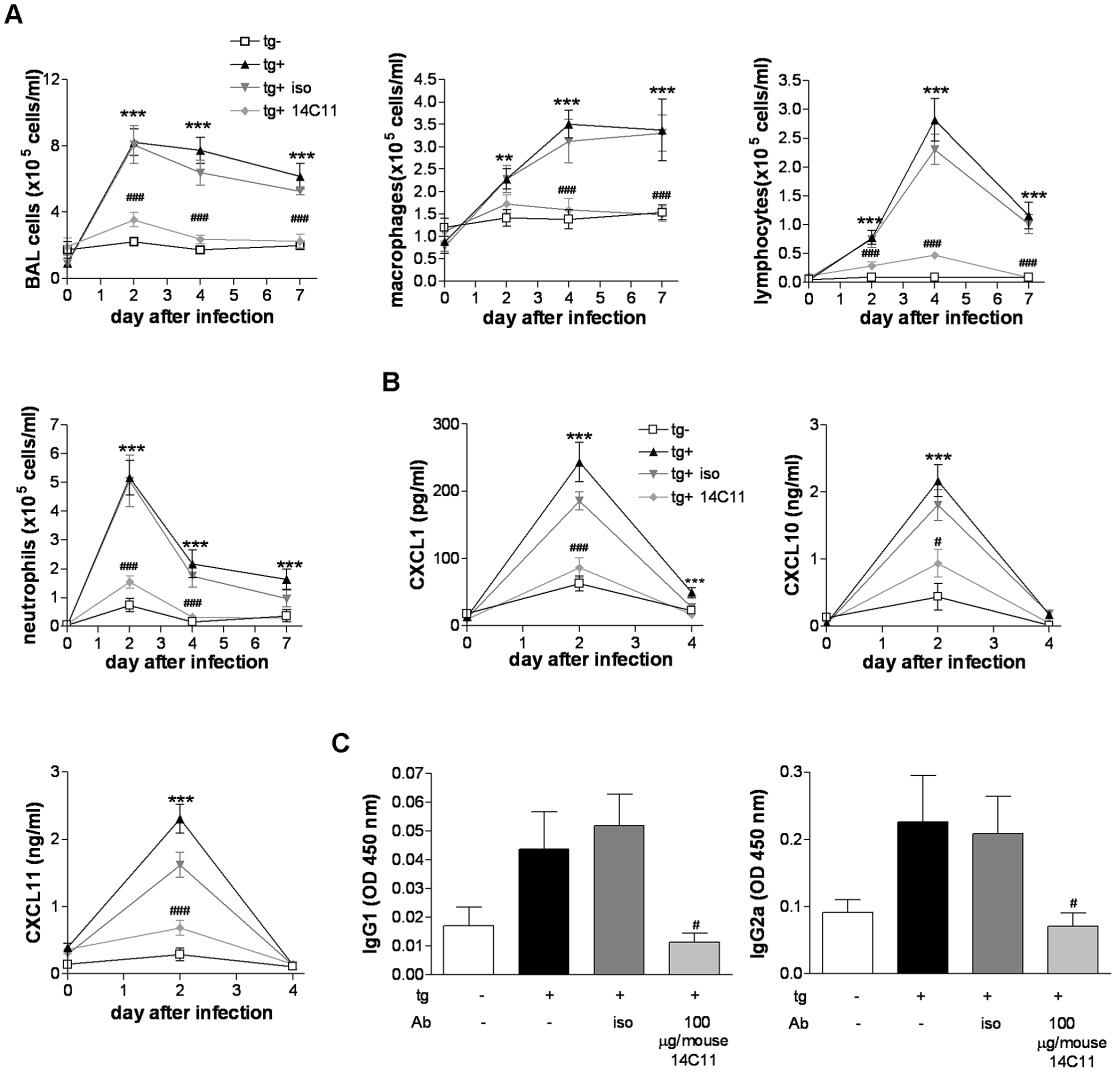
Time course of topically dosed 14C11 antibody-mediated inhibition of major group HRV16 infection. Mice were dosed intranasally with 14C11 or isotype control 2 hours prior to intranasal infection with HRV16. (A)Total BAL cells, macrophages, lymphocytes and neutrophils were assessed with differentially stained cytospins day 2, 4 and 7 after infection. (B) CXCL1, CXCL10 and CXCL11 in the BAL were determined by MSD or quantitative ELISA. (C) HRV16 specific serum IgG1 and IgG2a on day 7 post-infection. Data are a pool of 2 experiments with n = 4 mice per group each. Data are expressed as mean (± SEM). Significance was assessed by Three-way analysis of variance (A and B) or by One-way ANOVA with Bonferroni's Multiple Comparison test as post-test (C). **p<0.01 and ***p<0.001 vs HRV16 infected transgenic negative mice; ^#^p<0.05 and ^###^p<0.001 vs HRV16 infected transgenic positive mice.

We then asked whether the progression of inflammatory responses to viral infection at later time points is altered by 14C11 to shed light on whether 14C11 simply retards, or completely arrests HRV16 infection. Therefore, we dosed tg+ mice intranasally 2 hours prior to viral infection either with or without 100 µg/mouse 14C11 antibody or 100 µg/mouse isotype control and screened for airway inflammation at day 2, 4 and 7 post infection. Tg− mice received the same dose of HRV16 as all other groups (Figure 6). In HRV16 infected tg+ mice as well as in infected tg+ mice pre-treated with isotype control total BAL cell numbers peaked at day 2 after infection and stayed increased till day 7. Counts of total BAL cells in tg+ mice pre-treated with 14C11 revealed the same total BAL cell numbers as those observed in tg− mice challenged with HRV16 up to day 7. Macrophages and lymphocytes peaked at day 4 after infection for the tg+ positive HRV16 group and mice pre-treated with isotype control; however macrophage numbers showed a prolonged increase while lymphocyte numbers decreased after day 4 and returned close to baseline. 14C11 significantly reduced macrophage and lymphocyte numbers to levels found in tg− littermates challenged with HRV16. Neutrophils were also recruited and highest levels of neutrophils could be observed at day 2 post-infection in HRV16 infected tg+ mice or isotype-control pre-treated and HRV16 infected tg+ mice. A single dose of 14C11 significantly reduced the number of neutrophils to control levels found in challenged tg− mice, over a sustained period of 7 days (Figure 6A).

IL-1β, IL-6 and the chemokines CXCL1, CXCL11 and CXCL10 as well as IFNλ2/3 in the BAL peaked at day 2 after infection in HRV16 infected tg+ mice and those pre-treated with isotype control, whereas tg− littermates and 14C11 treated HRV16 infected tg+ mice failed to induce cytokine and chemokine production. All cytokines and chemokines returned to baseline levels on day 4 of infection, except IFNλ2/3 which could still be detected on day 4 in tg+ infected with HRV16 and isotype control treated animals, but not in tg− mice or tg+ mice pre-treated with 14C11 (Figure 6B and S4A). Similar results were found in lung homogenate for levels of IL-1β, IL-6 and CXCL1 protein (Figure S4B).

We next checked the influence of 14C11 on the adaptive immune response by measuring HRV16-specific antibodies. HRV16 infected tg+ and isotype control pre-treated mice showed increased levels of HRV16-specific IgG1 and IgG2a in serum on day 7 post-infection. Levels of HRV16 specific IgG1 and IgG2a were reduced in infected and 14C11 treated and in tg− HRV16 challenged mice (Figure 6C). A single dose of 14C11 was therefore able to decrease HRV16-induced cellular infiltration in the lung, induction of cytokines and chemokines, and the induction of HRV16-specific antibodies over a sustained period of 7 days.

### 14C11 pre-treatment prevents HRV16-induced exacerbation of allergic airway inflammation *in vivo*

**Figure 7:**
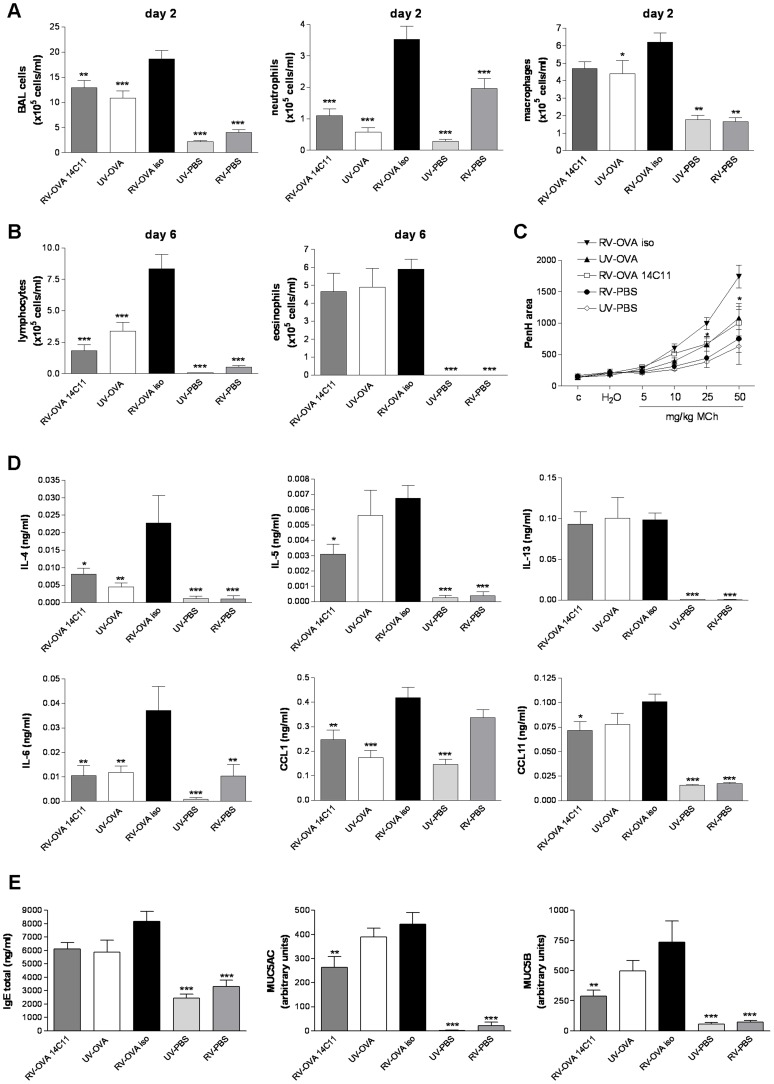
Effect of topically dosed 14C11 antibody on HRV16 induced exacerbation of allergic airway inflammation in vivo. Groups of 8 transgenic positive mice were OVA sensitised on day −13 and re-challenged either with PBS or OVA on day −2 and day −1. On day 0 mice were dosed intranasally with 14C11 or isotype control 2 hours prior to intranasal challenge with PBS or OVA and infected HRV16 (RV-PBS and RV-OVA) or UV-inactivated HRV16 (UV-PBS or UV-OVA). (A) Total BAL cells, neutrophil and macrophage numbers were assessed on day 2 after infection and (B) lymphocyte and eosinophil numbers on day 6 after infection. (C) Airway hyper-responsiveness was determined on day 1 after infection. The levels of cytokines and chemokines (D) IL-4, IL-5, IL-13, IL-6 and CXCL1 in BAL and CXCL11 in lung homogenate 2 days after infection. (E) Total IgE in serum and levels of MUC5AC and MUC5B protein in BAL on day 6 after infection. Significance was assessed by One-way ANOVA test with Bonferroni's Multiple Comparison test as post-test. *p<0.05, **p<0.01 and ***p<0.001 vs transgenic positive mice pretreated with isotype control and challenged with RV-OVA. Data are representative of 3 independent experiments.

Allergic asthma is exacerbated by viral infections, especially by HRV infections. To model virus-induced exacerbations of allergic airway inflammation, transgenic positive mice were sensitised and challenged with OVA, and infected with HRV16. We asked whether 14C11 could prevent HRV16-induced exacerbation of allergic airway inflammation. The asthma-like phenotype with eosinophilic airway inflammation was confirmed in all 3 groups challenged with OVA on day 6 after infection (Figure 7B). Mice not challenged with OVA, but HRV16 infected (RV-PBS) showed increased neutrophil numbers on day 2 compared to UV-HRV16 infected mice (UV-PBS). Compared to mice challenged with UV-inactivated HRV16 and OVA (UV-OVA), HRV16 and OVA challenge in isotype pre-treated tg+ mice (RV-OVA iso) significantly increased total BAL cell and neutrophil numbers on day 2 after infection, lymphocyte numbers on day 6 and increased airway hyper-responsiveness as measured by PenH at 24 hrs post-challenge. 14C11 pre-treated, HRV16 and OVA challenged mice (RV-OVA 14C11) showed reduced numbers of total BAL cells, neutrophils and lymphocytes (Figure 7A and B). RV-OVA-14C11 treatment also reduced the exacerbation of airway hyper-responsiveness to UV-OVA control levels on day 1 after infection (Figure 7C).

To further investigate the impact of 14C11 pre-treatment on HRV16-induced allergic airway inflammation we analysed Th2-type cytokine concentrations as well as the neutrophil and eosinophil chemokine attractants CXCL1 in BAL and eotaxin-1 levels in lung homogenate at day 2 after infection. Higher levels of IL-4, IL-5 and IL-6 were found in RV-OVA iso mice compared to mice receiving RV-OVA 14C11 treatment (Figure 7D). The level of IL-13 in OVA challenged groups was not increased by virus and therefore no effect of 14C11 treatment was observed. The neutrophil attractant CXCL1 was induced in all groups infected with HRV16 but not modulated by OVA. However, 14C11 administration reduced the level of CXCL1 in RV-OVA challenged animals. RV-OVA 14C11 treated mice exhibited a small but significant reduction in eotaxin-1 levels compared to RV-OVA iso treated controls (Figure 7D). Total IgE levels measured in serum were not significantly increased by virus infection and not altered by 14C11 pre-treatment.

Because in asthma exacerbations, increased inflammation and mucus production have been observed we analysed the impact of 14C11 on mucus production in HRV16-exacerbated allergic airway inflammation. RV-OVA iso challenged mice showed a small increase in the production of mucins MUC5AC and MUC5B compared to mice challenged with UV-OVA challenge. 14C11 pre-treatment of RV-OVA challenged mice significantly reduced the induction of both MUC5AC and MUC5B mucus proteins 6 days after infection (Figure 7E). Our data showed that neutralisation of the HRV16 entry receptor ICAM-1 by 14C11 reduced HRV16-induced allergic airway inflammation *in vivo*.

These data indicate the possibility to design a domain-specific anti-ICAM-1 antibody to specifically hinder major group HRV entry and replication without blocking the crucial LFA-1 function for unaltered cell recruitment to the site of infection. We have shown for the first time a major group allergen-induced asthma exacerbation model and the possible use of a domain-specific anti-ICAM-1 antibody as possible treatment for rhinoviral triggered exacerbations of asthma.

## Discussion

Human rhinovirus infection is the most common infection afflicting mankind and is responsible for enormous morbidity and societal costs as the major cause of the common cold. It also causes severe morbidity, mortality and health care costs as a key trigger of acute exacerbations of lung diseases such as asthma, COPD and cystic fibrosis. In this study, we demonstrate that an anti-ICAM-1 domain-specific antibody can effectively block entry and replication in an *in vivo* model of human rhinovirus infection and reduce rhinovirus-induced exacerbation of allergic airway inflammation, without preventing ICAM-1 interaction with its cellular ligand LFA-1, which is required for cell recruitment during respiratory infections. Thus 14C11 and other antibodies with similar specificities could be useful agents to help reduce rhinovirus induced respiratory exacerbations. .

The majority of HRVs use a single cellular receptor, ICAM-1, for attachment to cells and therefore ICAM-1 is an interesting target to block virus-receptor binding to prevent rhinovirus infection. Several alternative approaches have been investigated, but have not been taken further in clinical trials or had to be abandoned because of side effects. For example, soluble ICAM-1 (tremacamra, BIRR4) reduced the severity of experimental colds [26], but was not developed further potentially due to the expense of manufacturing recombinant proteins as drugs, as well as the frequency of dosing required. Interferon-alpha used intranasally was also shown to be effective against HRV infection, but had side effect including leukopenia and nasal bleeding [27]. The HRC 3C protease inhibitor (Ruprintrivir) was reported to reduce the proportion of subjects with positive viral cultures and viral titres but did not decrease the frequency of colds [28], [29]. Pleconaril has also been shown to be clinically effective in reducing the duration of colds, but did not get approval due to its side effects profile [30], [31]. Lack of cross-protection between different serotypes of HRV has also meant that no successful vaccine has been developed and thus no specific therapy against HRVs exists, even though there is a very high medical need.

Antibody therapy is an attractive alternative therapeutic approach, as a single injection can provide protection of long duration. In this present study we have identified a human ICAM-1 specific antibody, 14C11, which specifically binds to domain 1 of human ICAM-1 (Figure 1), the rhinovirus binding domain [32]. 14C11 was able to block infection *in vitro* with HRV16 and HRV14, members of the HRV A-type and B-type group, respectively. Moreover, 14C11 was able to block a wide range of other major group rhinovirus strains, suggesting that blocking of rhinovirus entry and replication with 14C11 could be seen as broad spectrum blocking agent for major group rhinoviruses (Figure 2).

ICAM-1, through its interaction with LFA-1 is also important in cell trafficking, and ICAM-1/LFA-1 interactions are reported to involved domains 1 of the ICAM-1 molecule [32], thus antibodies to ICAM-1 have theoretical potential to inhibit cell trafficking through blocking ICAM-1/LFA-1 interaction. Binding assays using the Jurkat human T cell line have shown that 14C11, in contrast to another anti-ICAM-1 antibody 84H10, did not interfere with LFA-1 interaction and therefore that this human ICAM-1 domain 1-specific antibody potentially only hinders HRV entry and replication (Figure 1).

To test the 14C11 antibody *in vivo* we infected transgenic mice over-expressing a chimeric ICAM-1 molecule, consisting of mouse ICAM-1 in which domains 1 and 2 were replaced with human ICAM-1 domains 1 and 2 [24], with the major group rhinovirus HRV16. We observed a cellular infiltration in the lung in HRV16 infected transgenic positive mice, comprising an increase in total BAL cells, lymphocytes and neutrophils, which was decreased in a dose dependent manner by topical administration of 14C11 for all parameters. Chemokines and cytokines in BAL and lung homogenate were also significantly reduced by 14C11 treatment. In addition, rhinovirus replication in transgene positive mice was completely blunted with 14C11 treatment, suggesting that blocking of all ICAM-1 binding sites in the lung can prevent HRV entry and replication *in vivo*. Histological analysis confirmed that treatment with 14C11 reduced inflammatory cell infiltration of the lung and was comparable to the lack of infiltration obtained by challenge of non-transgenic littermates with HRV16 (Figure 3).

Genome sequence places HRV16 in group A which also contains the majority of ICAM-utilising major group viruses (63 out of a currently identified 88 serotypes). HRV14 is amongst a significant minority of genetically distinct major group viruses (25 serotypes) that belong to group B viruses [17]. We could additionally show that infection with HRV14 could be prevented by 14C11 *in vivo*. Cellular infiltration in the lung, as well as cytokine and chemokine levels in BAL and lung homogenate were all reduced by topical administration of 14C11 (Figure S2). Thus ICAM-1 blockade has shown efficacy *in vivo* against representative serotypes of both species of major group rhinoviruses. Of the 99 serotyped rhinovirus strains, major group viruses which use ICAM-1 and which can potentially be specifically blocked by 14C11 constitute 88 serotypes, while minor group viruses, which do not use ICAM-1 and which therefore would not be treated with 14C11, constitute only 11 serotypes, thus 14C11 has potential to treat 89% of serotyped rhinovirus strains. A third group of rhinoviruses named group C viruses has been recently identified based on RNA genome sequence analysis [33]. The size of this group of rhinoviruses is estimated on sequence analysis to be around 60 virus strains. Viruses in this group have not been serotyped as they have not yet been successfully cultured in a cell line [16], however based on sequence analysis, it is assumed that this group of viruses will not use ICAM-1 as their cellular receptor [16]. If this is confirmed by experimental data antibodies targeting the same epitope to 14C11 and thus only inhibiting major group virus entry would be anticipated to block∼55% of rhinovirus strains known to date.

Therapeutic use of antibodies in man normally involves systemic rather than inhaled dosing, for this reason we wished to determine if systemic dosing with 14C11 would successfully inhibit rhinovirus induced airway inflammation. Systemic dosing of 14C11 did prevent HRV16 induced inflammation and proinflammatory cytokine release, thus showing pharmacological activity of 14C11 via the intravenous route and suggesting the suitability of parenteral dosing for route of administration for an anti-ICAM1 based therapy (Figure 4 and Figure S3).

To investigate the human ICAM-1-specificity of the action of 14C11 *in vivo* and to exclude possible interactions of the Fc part of the 14C11 antibodies we tested for possible inhibition by 14C11 in minor group HRV infection, which does not use human ICAM-1 as virus entry receptor and in an LPS challenge model where induction of airway inflammation is independent of human ICAM-1 (Figure 5 and Figure S3). In both models of airway inflammation, we found that 14C11, which does not bind mouse ICAM-1 *in vitro* (Figure 1), also had no effect on human ICAM-1 independent airway inflammation *in vivo*. These data confirmed that the inhibition of HRV infection and induction of airway inflammation *in vivo* was specifically a consequence of blockage of rhinovirus entry and replication, and not due to non-specific effects.

We next investigated whether a single administration of 14C11 prior to HRV infection was effective in significantly inhibiting later outcomes of HRV infection. Complete inhibition of HRV induced total cell counts, macrophages, lymphocytes and neutrophils as well as virus-specific antibody induction were achieved up to 7 days after administration of 14C11 (Figure 6), thus strengthening our hypothesis that domain specific blocking of HRV entry and replication could be a potential anti-viral approach in man. These data also confirmed that 14C11 treatment completely arrested, rather than simply retarded HRV infection *in vivo*.

Having shown that this antibody could inhibit rhinovirus infection and induction of inflammation *in vivo*, we next wished to test the antibody in a model of disease where the costs in man would justify the development of a relatively expensive treatment such as a therapeutic antibody. Rhinovirus infections are responsible for the majority of acute exacerbations of asthma and exacerbations are responsible for around 50% of asthma related health care costs [34]. We therefore tested the antibody in a mouse model of rhinovirus exacerbation of allergic airway inflammation [24]. We show that 14C11 was effective in suppressing each of the outcomes that were exacerbated by rhinovirus infection superimposed on a model of ovalbumin induced allergic airway inflammation, including HRV-induced exacerbation of total cellular, neutrophilic and lymphocytic airway inflammation, as well as Th2 (IL-4 and IL-5) and pro-inflammatory (IL-6) cytokine and neutrophil (CXCL1) and eosinophil (eotaxin) recruiting chemokine release. Additionally, 14C11 treatment significantly suppressed HRV exacerbation of the two major respiratory mucins MUC5AC and MUC5B, and importantly, airway hyper-responsiveness.

In conclusion we have shown that the human ICAM-1 domain 1-specific antibody 14C11 inhibits major group HRV infection *in vitro* and *in vivo*, as well as inhibiting major group HRV-induced exacerbation of allergic airway inflammation *in vivo*. This antibody however did not interfere with human ICAM-1 binding to its ligand on human T cells and further it had no non-specific effects on cell recruitment in models of airway inflammation induced by mechanisms independent of human ICAM-1. This antibody and others with similar properties would therefore be good candidates for development of novel treatments for diseases induced by major group rhinoviruses – principally acute exacerbations of airway diseases such as asthma, COPD and cystic fibrosis.

## Materials and Methods

### Ethics statement

All animal work was completed in accordance with UK Home Office guidelines following approval via the ethical approval process (UK project licence PPL 70/7234 valid 03/03/2011 to 03/03/2016).

### ICAM specificity ELISA

ICAM-1 antigens were generated using mammalian expression systems. Human ICAM-1 domain 1/mouse ICAM-1 domains 2–5 chimera, and mouse ICAM-1 domains 1–5 were generated in-house. Human ICAM-1 domains 1–5 was obtained commercially (R&D Systems 720-IC). All ICAM-1 antigens were biotinylated via free amines using EZ link Sulfo-NHS-LC-Biotin (Thermo Scientific). Biotinylated bovine insulin (Sigma I2258) was used as control for non-specific binding. Streptavidin plates (Thermo Scientific) were coated with the biotinylated antigens at 1 µg/ml in PBS and incubated overnight at 4°C. Plates were washed, blocked (PBS+0.1% BSA) for 1 hour and serial dilutions of anti-ICAM-1 antibody 14C11 (MAB720, R&D Systems) were added in blocking buffer for 1 hour as indicated in the figures. ELISA to test 14C11 binding specificity used 14C11 and control antibodies from R&D Systems: anti-mouse ICAM-1 (MAB796) anti-human ICAM-2 (MAB244), anti-human ICAM-3 (MAB813), anti-human ICAM-5 (MAB1173) and anti-human VCAM-1 (MAB809). Plates were washed and developed with anti-human IgG light chain HRP antibody (Sigma), TMB and H_2_SO_4_. Plates were read on an EnVision™ plate reader at 450 nm.

### PMA Jurkat cell adhesion assay

Human ICAM-1-Fc (R&D Systems) was coated onto 96-well black walled plates at 10 µg/ml in PBS overnight at 4°C. Plates were then washed and blocked with assay buffer (phenol-red free HBSS plus 1% BSA) for 1 h. Antibodies (14C11, 84H10 (Serotec) and muIgG1 isotype control were serially diluted in assay buffer (4× final concentration) and 50 µl/well transferred to ICAM-1-Fc coated plates. After a 15 min incubation period at room temperature, a further 50 µl/well of PMA (50 ng/ml) in assay buffer was then added. During this time, Jurkat E6.1 cells were harvested and labelled with Calcein-AM dye (5 µM in phenol-red free RPMI 1640) for 30 minutes at 37°C. Following washing, 10^5^ Jurkat E6.1 cells were added to each well in 100 µl assay buffer. Plates were then incubated for a further 3 h at 37°C/5% CO_2_. Adhesion of the cells to the plate was assessed following aspiration of the plate and washing with 250 µl wash buffer (150 mM HEPES, 0.1% glucose, 2 mM MgCl_2_ pH 7.2). A final 250 µl of wash buffer was added and plates were read for fluorescein detection. Data are expressed without PMA (0% signal) and with PMA and no antibody (100% signal).

### Cytopathic effect assay

HeLa Ohio cells (ECACC) were seeded at 3×10^4^/well into 96 well plates in MEM (minimal essential medium) supplemented with 1× non-essential amino acids, penicillin (100 U/ml), streptomycin (100 µg/ml) (each from Invitrogen, Paisley, UK) and heat inactivated bovine calf serum (10%), (SAFC Biosciences, Andover, UK). After an overnight incubation (37°C/5% CO_2_) the media was removed and replaced with serially diluted anti-ICAM-1 antibody 14C11, mIgG1 isotype control (R&D Systems, Abingdon) or medium alone for untreated controls and incubated at 37°C for 30 minutes. Following this, 30 µl of HRV16 or HRV14 culture supernatant (multiplicity of infection predetermined to induce 80–90% CPE) was then added to the wells (or medium alone for un-infected controls) and virus attachment was allowed to proceed for 2 hours at 33°C/5% CO_2_. Assay supernatants were then removed and 100 µl of fresh medium was added to each well and incubated for a further 23 hours at 33°C/5% CO_2_. Remaining viable cells were fixed in 4% formaldehyde (Sigma-Aldrich, Poole, UK), stained with 0.5% w/v crystal violet (Sigma-Aldrich, Poole, UK) for 5 minutes, washed 3 times in water to remove excess stain and allowed to dry. The extent of CPE was quantified by measuring absorbance at 600 nm and calculated according to the equation % CPE = 100×[(test sample−virus only)/(uninfected control−virus only)].

### Mice

Human-mouse ICAM-1 transgenic mice were generated as described previously [24]. Human-mouse ICAM-1 transgenics were bred in-house and transmission of the chimeric transgene was confirmed by PCR. Mice bearing the human-mouse ICAM-1 transgene are referred in the paper as tg+ and mice not expressing the transgene are referred as tg−.

### Viruses

HRV were grown in HeLa Ohio cells. Infected cells were harvested after 24 hours and HRV was concentrated and purified as described previously [24]. Viral titers were assessed by TCID_50_ assay (50% tissue culture infectivity dose). The identity of HRV1B, HRV14 and HRV16 working stocks were confirmed by neutralisation with serotype specific antibodies (ATCC). The HRV infection and HRV-induced asthma exacerbation models have been described previously [24]. For infection studies mice were infected with 5×10^6^ TCID_50_/ml of the indicated HRV. For the mouse asthma exacerbation model mice were infected with 2.5×10^6^ TCID_50_/ml. To demonstrate HRV replication-specific responses HRV1B and HRV16 were UV-irradiated using a UV Stratalinker 2400 (Stratagene).

### Human rhinovirus infection model

Mouse anti-human ICAM-1 antibody 14C11 (MAB720, R&D Systems) and mouse IgG1 isotype control (MAB002, R&D Systems) was used as a 0.2 µm filtered solution dissolved in PBS. 5 to 7 week old transgenic positive (tg+) or transgenic negative (tg−) females were lightly anaesthetised and dosed intranasally with mouse anti-human ICAM-1 antibody 14C11 or isotype control 2 hours prior to challenge with HRV16 or HRV14. For intravenous treatment, 14C11 or isotype control was administered in the tail vein 24 hours before infection with HRV16, HRV1B or UV-inactivated HRV1B (UV-HRV1B). Bronchoalveolar lavage (BAL) was performed using 1.5 ml BAL buffer (EBSS, 55 mM EDTA, 12 mM lidocaine) 1, 2, 4 or 7 days after infection, as indicated. BAL supernatant was aliquoted and stored for cytokine and chemokine analysis. BAL cells were counted and processed onto slides by cytospin, differentially stained (Quick Diff, Reagena, Finland) and counted blind to experimental conditions. 300 cells were counted per slide. The small upper lung lobe was stored in RNAlater (Qiagen) for mRNA analysis. The remaining lung tissue was homogenised in 2 ml of PBS, clarified by centrifugation and stored for cytokine and chemokine analysis.

### HRV-induced asthma exacerbation model

5 to 7 week old transgenic positive females were injected intraperitoneally (i.p.) with 50 µg OVA (Albumin from chicken egg, Calbiochem) and 2 mg aluminium hydroxide (Sigma) in a volume of 200 µl on day −13. Lightly anesthetised mice were challenged with 40 µg OVA/mouse on day −2 and day −1 to induce allergic airway inflammation. On day 0 anesthetised mice were challenged either with 20 µg OVA or PBS and infected with HRV16 or dosed with UV-inactivated HRV16 (RV-OVA, UV-OVA or RV-PBS, UV-PBS). Airway hyper-reactivity (AHR) was assessed by whole body plethysmography on day 1 after infection in response to increasing doses of nebulised methacholine (MCh). BAL supernatant and cells were collected at day 2 or day 6 after infection and processed as described above.

### Quantification of cytokines and chemokines, IgE, mucus and tissue infiltration

Cytokines and chemokines in BAL or lung homogenate were measured by ELISA using commercial kits from R&D Systems or Meso Scale Discovery. IL-4, IL-5 and IL-13 were measured with R&D Quantikine ELISA kits; eotaxin, IP-10, ITAC and IFNλ2/3 were measured with R&D Duosets; IL-1β, IL-6 and CXCL1 were measured with MSD multi-array according to the manufacturer's recommendations. Total IgE was measured in serum with BD OptEIA (BD Bioscience) according to the manufacturer's instructions. Mucins MUC5AC and MUC5B were determined by semiquantiative ELISA: BAL fluid was diluted in PBS and dried over night in Maxisorp plates (Nunc). MUC5AC was detected using a biotinylated anti-MUC5AC antibody (Neomarkers) and detected with streptavidin-HRP (Invitrogen) and TMB (Sigma). MUC5B was detected using an anti-MUC5B antibody (Santa Cruz Biotechnology) and developed using a biotinylated anti-mouse IgG antibody (Sigma), streptavin-HRP and TMB. Lung tissues were freshly fixed in 10% buffered formaldehyde, embedded in paraffin wax, sectioned 5 µm thick and stained with haematoxylin-eosin (H&E). The morphology of H&E stained sections were visualized and images acquired using an Axioskop 40 light microscope with AxiCamMRc 5 digit camera (Zeiss, West Germany).

### HRV-specific antibody enzyme-linked immunosorbent assay

Nunc Maxisorp Immuno plates were coated with purified HRV stock in PBS overnight. After blocking, diluted serum samples were incubated for 1 hour, followed by detection with biotinylated rat-anti-mouse IgG1 or IgG2a antibodies. The assay was developed with streptavidin-HRP and TMB as substrate.

### LPS challenge model

Transgenic positive mice were dosed intravenously with 14C11 24 hours prior to intranasal challenge with 1 µg LPS/mouse. Lipopolysaccharide (LPS) from E. coli 055:B5 was purchased from Sigma. BAL was collected and processed as described above.

### Quantitative real-time PCR

The left upper lung lobe was stored in RNAlater (Qiagen) until RNA was purified using the RNeasy Mini kit (Qiagen) and the RNase-free DNase set (Qiagen). Isolated RNA was reverse-transcribed using random hexamer primers (OmniscriptRT kit, Qiagen). Real-time PCR was performed with the Quantitect Probe PCR master mix (Qiagen). Primers and probes for 18S rRNA (forward: 5′cgc cgc tag aggtgaaattct; revers: 5′cat tcttggcaaatgctttcg and probe: FAM5′acc ggcgcaagacggaccaga) and HRV specific primers (forward: 5′ gtgaagagccscrtgtgc t; reverse: gctscagggttaaggttagcc and probe FAM5′tga gtcctccgg ccc ctgaat g) were used for quantification of HRV16 vRNA.

### Statistical analysis

All data were distributed normally and data are expressed as means ± SEM. Data were analysed with Prism3 (Graph Pad) or SAS Version 8.

## Supporting Information

Figure S1 **The antibody 14C11 is specific for domain 1 of human ICAM1 and does not cross-react with other ICAM family members.** To determine antibody specificity 14C11 was tested for binding to a panel of ICAM family members. Using an ELISA 14C11 was incubated with human (Hu) ICAM1, Mouse ICAM1, Hu ICAM-2, -3, -5 and Hu VCAM1. In addition a chimeric protein consisting of human ICAM1 domain 1 (Hu D1) and mouse domains 2 to 5 (Mu D2-5) was evaluated. Specific anti-ICAM-2, -3, -5 and VCAM1 antibodies were used as positive controls. Recombinant insulin was also used as a negative control to determine non-specific binding.
(TIF)

Figure S2 **Topically dosed 14C11 antibody inhibits HRV14 induced inflammation.** Groups of 6 mice were dosed intranasally with 14C11 2 hours prior to intranasal infection with HRV14. (A) Total BAL cells, macrophages, lymphocytes and neutrophils were assessed with differentially stained cytospins day 2 after infection. (B) The levels of proinflammatory cytokines and chemokines IL-1β, IL-6, CXCL1, IFNλ3, CXCL11 and CXCL10 in BAL were determined by MSD or quantitative ELISA 2 days after infection. (C) The levels of proinflammatory cytokines and chemokines IL-1β, IL-6 and CXCL1 in lung homogenate were assessed with MSD 2 days after infection. Data are expressed as mean (± SEM). Significance was assessed by One-way ANOVA test with Bonferroni's Multiple Comparison test as post-test. *p<0.05, **p<0.01 and ***p<0.001 vs HRV14 infected transgenic negative mice; ^##^p<0.01 and ^###^p<0.001 vs HRV14 infected transgenic positive mice. Data are representative of 2 independent experiments.
(TIF)

Figure S3 **Systemically dosed 14C11 antibody inhibits HRV16 induced inflammation.** Mice were dosed intravenously with 14C11 24 hours prior to intranasal infection with HRV16 (n = 9 for tg− group; n = 6 for tg+ groups). (A) The levels of proinflammatory cytokines IL-1β, IL-6, IFNλ2/3 were determined in BAL by MSD or quantitative ELISA. (B) The levels of proinflammatory cytokines and chemokines IL-1β, IL-6 and CXCL1 in lung homogenate were assessed with MSD. Data are expressed as mean (± SEM). Significance was assessed by One-way ANOVA test with Bonferroni's Multiple Comparison test as post-test. **p<0.01 and ***p<0.001 vs HRV16 infected transgenic negative mice; ^#^p<0.05, ^##^p<0.01 and ^###^p<0.001 vs HRV16 infected transgenic positive mice; Data are representative of 3 independent experiments.
(TIF)

Figure S4 **Systemically dosed 14C11 antibody specifically inhibits major group HRV induced inflammation.** Mice were dosed intravenously with 14C11 24 hours prior to intranasal infection with minor group HRV1B, UV-inactivated HRV1B (UV) or major group HRV16 (n = 4 for tg− UV, tg− 1B, tg+ 16 iso and tg+ 16 14C11 groups; n = 6 for tg+ UV, tg+ 1B, tg+ 1B iso and tg+ 1B 14C11 groups). (A) Total BAL cells, macrophages, neutrophils (day 1 p.i.) and lymphocytes (day 4 p.i.) were assessed with differentially stained cytospins. (B) The levels of proinflammatory cytokines IL-1β, IL-6, CXCL1 and IFNλ3 in BAL were determined by MSD or quantitative ELISA day 1 after infection. (C) The levels of proinflammatory cytokines and chemokines IL-1β, IL-6 and CXCL1 in lung homogenate were assessed with MSD day 1 after infection. Data are expressed as mean (± SEM). Significance was assessed by One-way ANOVA test with Bonferroni's Multiple Comparison test as post-test. *p<0.05, **p<0.01 and ***p<0.001 vs UV-RV1B in transgenic negative mice; ^#^p<0.05, ^##^p<0.01 and ^###^p<0.001 vs UV-RV1B in transgenic positive mice; ^§^p<0.05 and ^§§§^p<0.001 vs isotype treated HRV16 infected transgenic positive mice. Data are representative of 2 independent experiments. Groups of 7 mice were dosed intravenously with 14C11 24 hours prior to intranasal infection with 1 µg LPS/mouse. (D) The levels of proinflammatory cytokines IL-1β, IL-6 and IFNλ3 in BAL were determined by MSD or quantitative ELISA day 1 after infection. (E) The levels of proinflammatory cytokines and chemokines IL-1β, IL-6 and CXCL1 in lung homogenate were assessed with MSD day 1 after infection. Data are expressed as mean (± SEM). Significance was assessed by One-way ANOVA test with Bonferroni's Multiple Comparison test as post-test. *p<0.05, **p<0.01 and ***p<0.001 vs transgenic positive mice without treatment. Data are representative of 2 independent experiments.
(TIF)

Figure S5 **Time course of topically dosed 14C11 antibody in major group HRV16 infection model.** Mice were dosed intranasally with 14C11 or isotype control 2 hours prior to intranasal infection with HRV16. (A) The levels of proinflammatory cytokines IL-1β, IL-6, IFNλ2/3 were determined in BAL by MSD or quantitative ELISA. (B) The levels of proinflammatory cytokines and chemokines IL-1β, IL-6 and CXCL1 in lung homogenate were assessed with MSD. Data are expressed as mean (± SEM). Significance was assessed by Three-way analysis of variance. **p<0.01 and ***p<0.001 vs HRV16 infected transgenic negative mice; ^#^p<0.05, ^##^p<0.01 and ^###^p<0.001 vs HRV16 infected transgenic positive mice. Data are a pool of 2 experiments with n = 4 mice per group each.
(TIF)
