# Supplementary figures and images for: An Anti-Human ICAM-1 Antibody Inhibits Rhinovirus-Induced Exacerbations of Lung Inflammation
Source: PLoS Pathog. 2013 Aug 1;9(8):e1003520. doi: 10.1371/journal.ppat.1003520 (PMC3731244; doi:10.1371/journal.ppat.1003520)

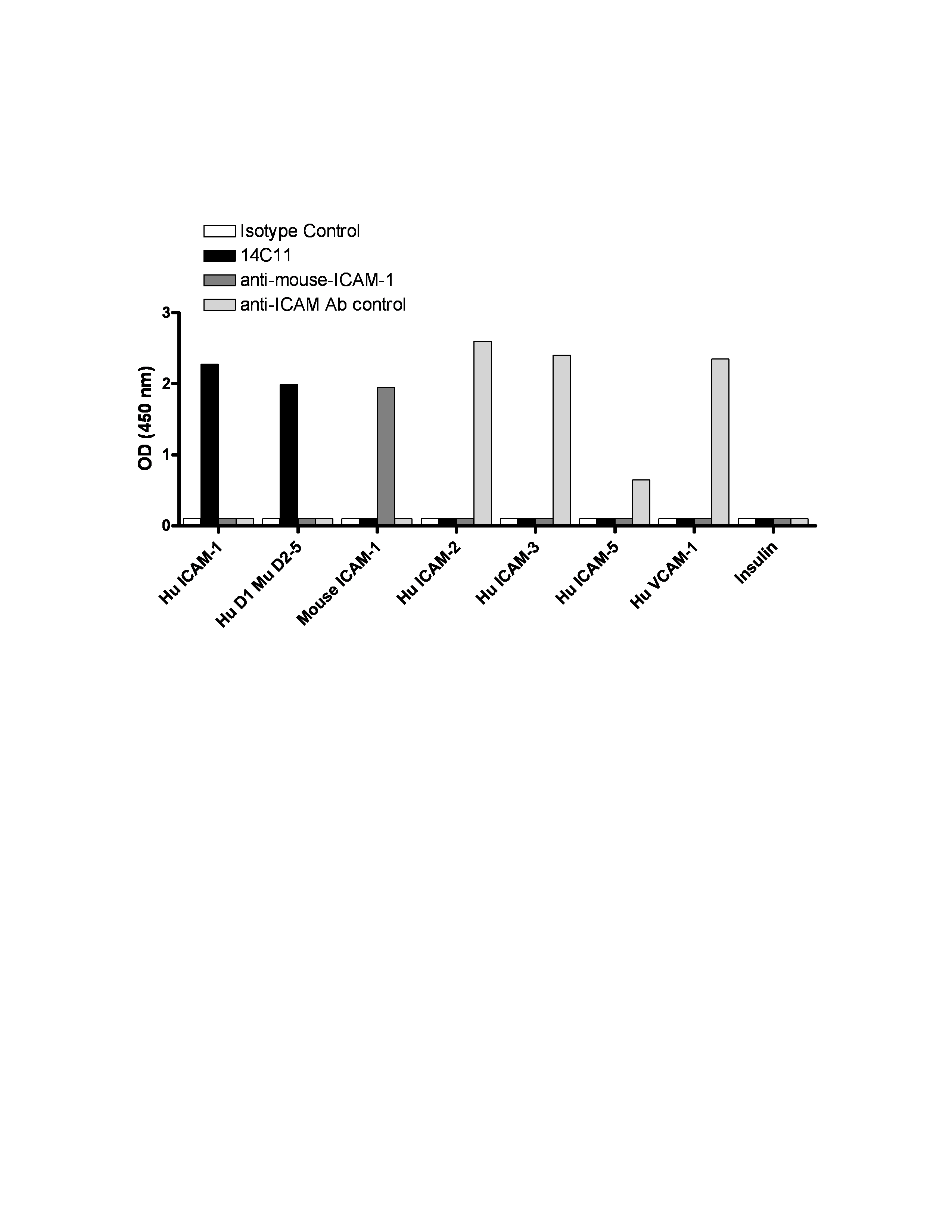

Supplement: Figure S1 — The antibody 14C11 is specific for domain 1 of human ICAM1 and does not cross-react with other ICAM family members. To determine antibody specificity 14C11 was tested for binding to a panel of ICAM family members. Using an ELISA 14C11 was incubated with human (Hu) ICAM1, Mouse ICAM1, Hu ICAM-2, -3, -5 and Hu VCAM1. In addition a chimeric protein consisting of human ICAM1 domain 1 (Hu D1) and mouse domains 2 to 5 (Mu D2-5) was evaluated. Specific anti-ICAM-2, -3, -5 and VCAM1 antibodies were used as positive controls. Recombinant insulin was also used as a negative control to determine non-specific binding. (TIF) [file ppat.1003520.s001.tif]

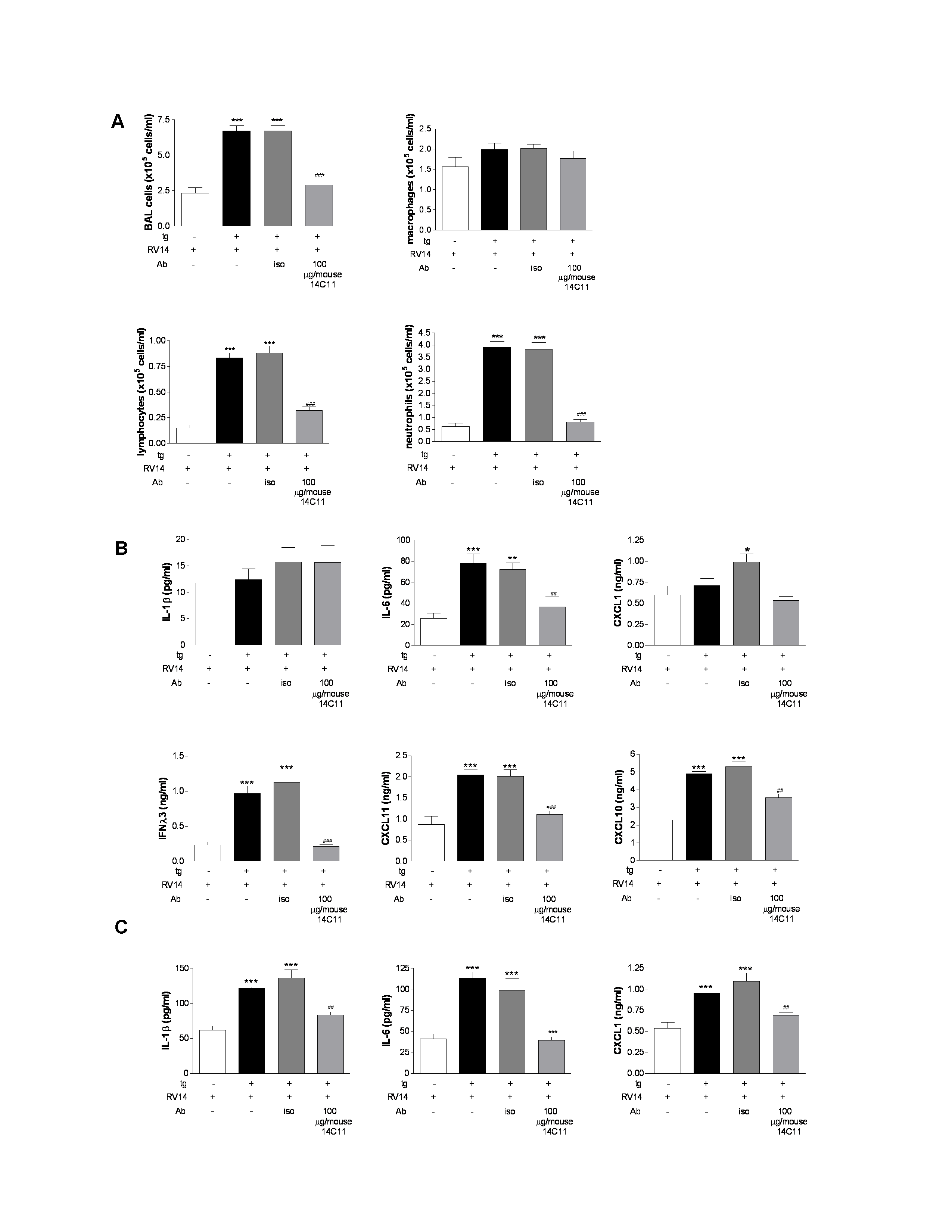

Supplement: Figure S2 — Topically dosed 14C11 antibody inhibits HRV14 induced inflammation. Groups of 6 mice were dosed intranasally with 14C11 2 hours prior to intranasal infection with HRV14. (A) Total BAL cells, macrophages, lymphocytes and neutrophils were assessed with differentially stained cytospins day 2 after infection. (B) The levels of proinflammatory cytokines and chemokines IL-1β, IL-6, CXCL1, IFNλ3, CXCL11 and CXCL10 in BAL were determined by MSD or quantitative ELISA 2 days after infection. (C) The levels of proinflammatory cytokines and chemokines IL-1β, IL-6 and CXCL1 in lung homogenate were assessed with MSD 2 days after infection. Data are expressed as mean (± SEM). Significance was assessed by One-way ANOVA test with Bonferroni's Multiple Comparison test as post-test. *p<0.05, **p<0.01 and ***p<0.001 vs HRV14 infected transgenic negative mice; ##p<0.01 and ###p<0.001 vs HRV14 infected transgenic positive mice. Data are representative of 2 independent experiments. (TIF) [file ppat.1003520.s002.tif]

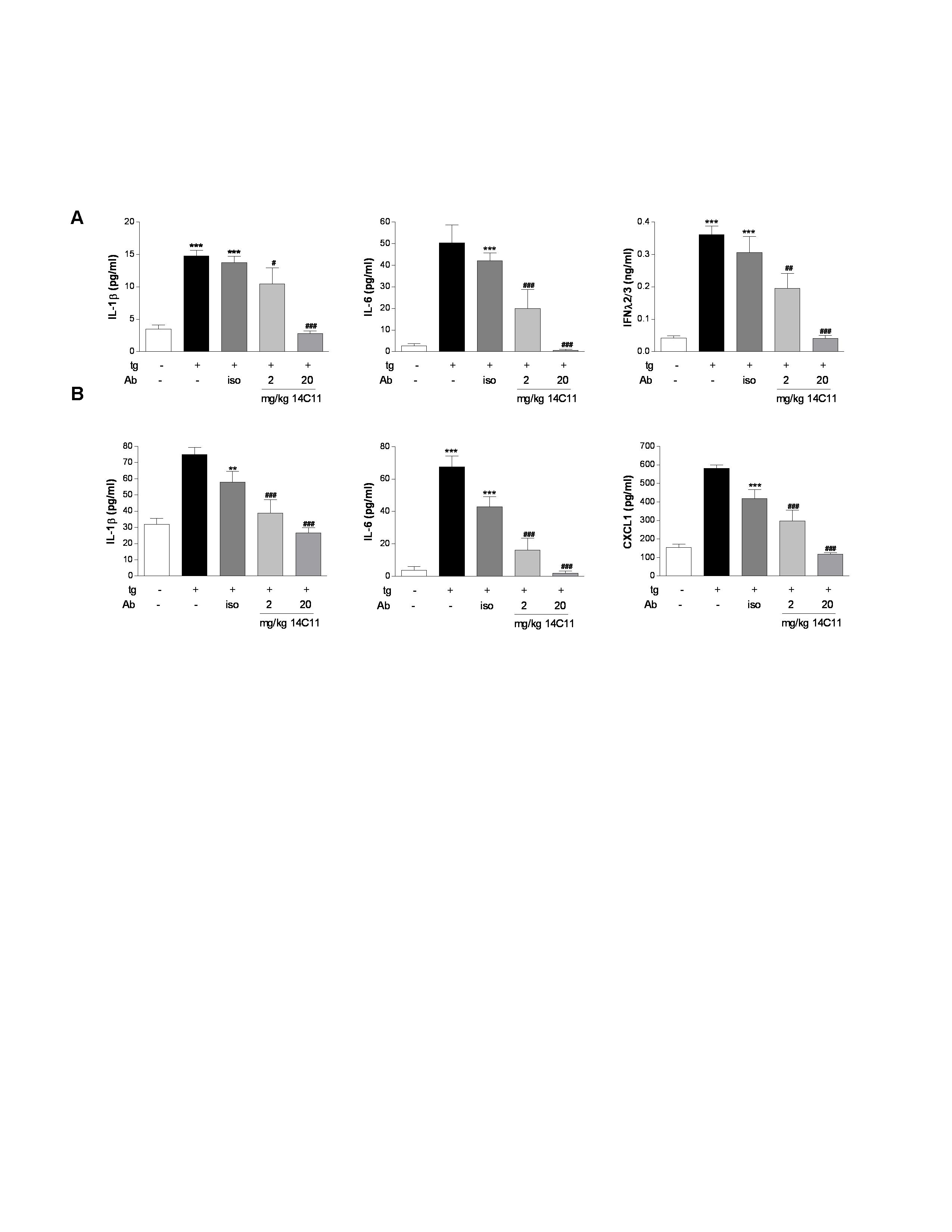

Supplement: Figure S3 — Systemically dosed 14C11 antibody inhibits HRV16 induced inflammation. Mice were dosed intravenously with 14C11 24 hours prior to intranasal infection with HRV16 (n = 9 for tg− group; n = 6 for tg+ groups). (A) The levels of proinflammatory cytokines IL-1β, IL-6, IFNλ2/3 were determined in BAL by MSD or quantitative ELISA. (B) The levels of proinflammatory cytokines and chemokines IL-1β, IL-6 and CXCL1 in lung homogenate were assessed with MSD. Data are expressed as mean (± SEM). Significance was assessed by One-way ANOVA test with Bonferroni's Multiple Comparison test as post-test. **p<0.01 and ***p<0.001 vs HRV16 infected transgenic negative mice; #p<0.05, ##p<0.01 and ###p<0.001 vs HRV16 infected transgenic positive mice; Data are representative of 3 independent experiments. (TIF) [file ppat.1003520.s003.tif]

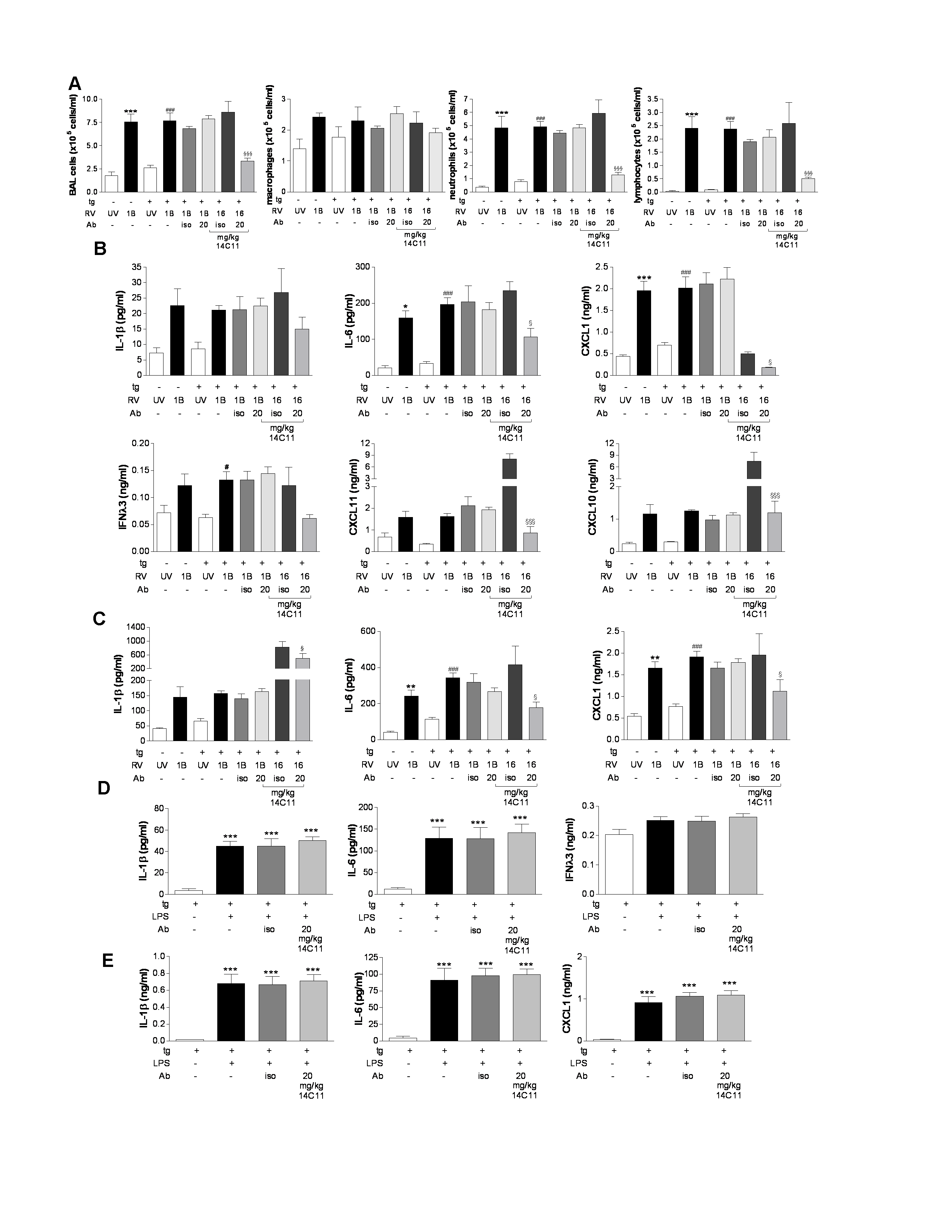

Supplement: Figure S4 — Systemically dosed 14C11 antibody specifically inhibits major group HRV induced inflammation. Mice were dosed intravenously with 14C11 24 hours prior to intranasal infection with minor group HRV1B, UV-inactivated HRV1B (UV) or major group HRV16 (n = 4 for tg− UV, tg− 1B, tg+ 16 iso and tg+ 16 14C11 groups; n = 6 for tg+ UV, tg+ 1B, tg+ 1B iso and tg+ 1B 14C11 groups). (A) Total BAL cells, macrophages, neutrophils (day 1 p.i.) and lymphocytes (day 4 p.i.) were assessed with differentially stained cytospins. (B) The levels of proinflammatory cytokines IL-1β, IL-6, CXCL1 and IFNλ3 in BAL were determined by MSD or quantitative ELISA day 1 after infection. (C) The levels of proinflammatory cytokines and chemokines IL-1β, IL-6 and CXCL1 in lung homogenate were assessed with MSD day 1 after infection. Data are expressed as mean (± SEM). Significance was assessed by One-way ANOVA test with Bonferroni's Multiple Comparison test as post-test. *p<0.05, **p<0.01 and ***p<0.001 vs UV-RV1B in transgenic negative mice; #p<0.05, ##p<0.01 and ###p<0.001 vs UV-RV1B in transgenic positive mice; §p<0.05 and §§§p<0.001 vs isotype treated HRV16 infected transgenic positive mice. Data are representative of 2 independent experiments. Groups of 7 mice were dosed intravenously with 14C11 24 hours prior to intranasal infection with 1 µg LPS/mouse. (D) The levels of proinflammatory cytokines IL-1β, IL-6 and IFNλ3 in BAL were determined by MSD or quantitative ELISA day 1 after infection. (E) The levels of proinflammatory cytokines and chemokines IL-1β, IL-6 and CXCL1 in lung homogenate were assessed with MSD day 1 after infection. Data are expressed as mean (± SEM). Significance was assessed by One-way ANOVA test with Bonferroni's Multiple Comparison test as post-test. *p<0.05, **p<0.01 and ***p<0.001 vs transgenic positive mice without treatment. Data are representative of 2 independent experiments. (TIF) [file ppat.1003520.s004.tif]

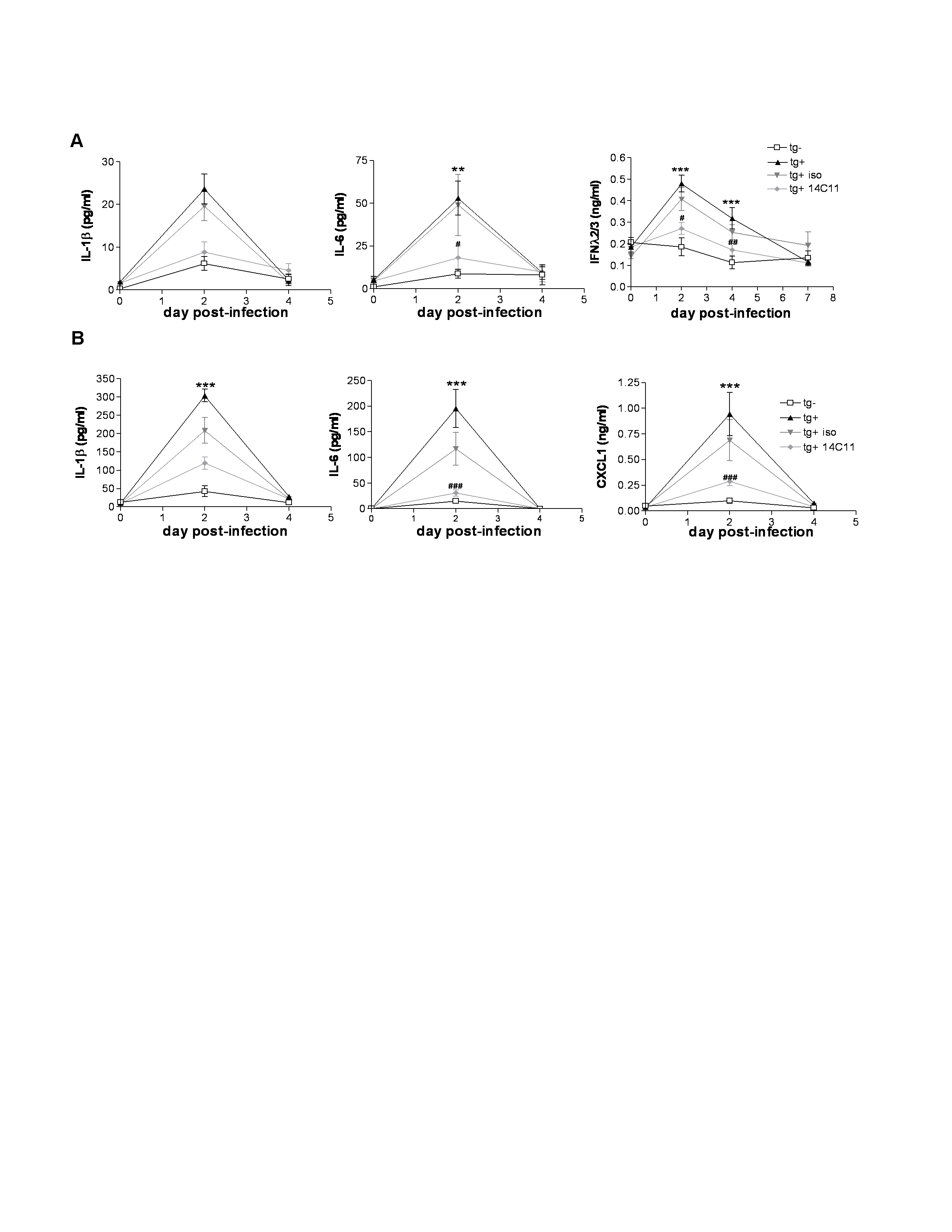

Supplement: Figure S5 — Time course of topically dosed 14C11 antibody in major group HRV16 infection model. Mice were dosed intranasally with 14C11 or isotype control 2 hours prior to intranasal infection with HRV16. (A) The levels of proinflammatory cytokines IL-1β, IL-6, IFNλ2/3 were determined in BAL by MSD or quantitative ELISA. (B) The levels of proinflammatory cytokines and chemokines IL-1β, IL-6 and CXCL1 in lung homogenate were assessed with MSD. Data are expressed as mean (± SEM). Significance was assessed by Three-way analysis of variance. **p<0.01 and ***p<0.001 vs HRV16 infected transgenic negative mice; #p<0.05, ##p<0.01 and ###p<0.001 vs HRV16 infected transgenic positive mice. Data are a pool of 2 experiments with n = 4 mice per group each. (TIF) [file ppat.1003520.s005.tif]
